# Resilience to COVID-19-related stressors: Insights from emerging adults in a South African township

**DOI:** 10.1371/journal.pone.0260613

**Published:** 2021-12-21

**Authors:** Linda Theron, Diane Levine, Michael Ungar

**Affiliations:** 1 Department of Educational Psychology, University of Pretoria, Pretoria, South Africa; 2 Leicester Institute of Advanced Studies, University of Leicester, Leicester, United Kingdom; 3 Resilience Research Center, Dalhousie University, Halifax, Canada; Neijiang Normal University, CHINA

## Abstract

There is widespread recognition that stressors related to Coronavirus Disease 2019 (COVID-19) jeopardize the development of emerging adults, more particularly those living in disadvantaged communities. What is less well understood is what might support emerging adult resilience to COVID-19-related stressors. In response, this article reports a 5-week qualitative study with 24 emerging adults (average age: 20) living in a South African township. Using digital diaries and repeated individual interviews, young people shared their lived experiences of later (i.e., month 4 and 7) lockdown-related challenges (i.e., contagion fears; livelihood threats; lives-on-hold) and how they managed these challenges. An inductive thematic analysis showed that personal and collective compliance, generous ways-of-being, and tolerance-facilitators enabled emerging adult resilience to said challenges. Importantly, these resilience-enablers drew on resources associated with multiple systems and reflected the situational and cultural context of the township in question. In short, supporting emerging adult resilience to COVID-19-related stressors will require contextually aligned, multisystemic responses.

## Introduction

On 11 March 2020, the World Health Organisation announced a Coronavirus Disease 2019 (COVID-19) pandemic. Their announcement signalled that countries must actively manage extensive community transmission of the Severe Acute Respiratory Syndrome Coronavirus 2 [1]. In response, the South African (SA) government declared a national state of disaster on 15 March 2020 [2]. Doing so facilitated a country-wide lockdown and raft of related mandatory regulations and disease mitigation strategies. That lockdown, described as “one of the most rigid and extreme lockdowns announced anywhere in the world” [3], curbed the spread of the coronavirus. Still, it was associated with widespread psychological and economic suffering that was skewed towards South Africa’s most disadvantaged people [4]. Rather than focus on that suffering, the objective of this article is to report how 24 African emerging adults managed the psychological and economic risks of lockdown (July 2020 –October 2020). In so doing, the article not only underscores the human capacity for resilience, but also responds to calls to better understand human resilience to COVID-19-related stressors [5]. More specifically, it responds to calls to better understand the resilience of emerging adults (18-29-year-olds [6]), given their pronounced vulnerability to COVID-19-related stressors [7–11].

### COVID-19-related challenges to emerging adult wellbeing

Even though emerging adults are less likely to contract COVID-19 (compared with older adults), their developmental stage makes them particularly vulnerable to COVID-19-related stressors [10, 12, 13]. Pronounced amongst these, are the economic stressors associated with pandemic-related lockdowns (e.g., loss of employment, decreased remuneration, material deprivation) [14], and how these jeopardise emerging adults’ ability to complete their education/training, establish a career, commit to a long-term partner, or achieve functional independence [6, 10, 11]. These core developmental tasks are similarly jeopardised by educational institution closures, restricted travel, and other lockdown-related disruptions [8]. There are also widespread concerns about psychological distress in the face/aftermath of lockdown-related isolation, domestic violence, or stressed household functioning, and/or fear for their own/significant others’ health [15–19].

### Emerging adult resilience to COVID-19-related challenges

Human resilience, or successful adjustment to significant stress, has been variably defined [20]. Nonetheless, systemic understandings have come to dominate the resilience literature [21, 22]. A systemic approach to resilience informed the study reported in this article.

Understood systemically, human resilience is a process that draws on resources distributed across multiple systems, including biological, psychological, social, and built/natural environment ones [23]. Which multisystem resources are more, or less, pivotal to the resilience process is specific to the risk in question and to a given situational and cultural context [21, 23, 24]. For instance, extended family resources are common to the resilience of young people from collectivist cultures, whereas a robust sense of self typifies the resilience of those from more individualistic cultures [25]. Guidelines designed to advance human resilience to COVID-19-related stresses reflect systemic understandings, and advocate for individuals/families to take action that will protect their wellbeing [26].

The emerging adult resilience literature, albeit limited, reflects this systemic understanding. We identified a single systematic review of emerging adult resilience studies [27]. That review concluded that biological and psychological resources (e.g., executive functioning skills; goal orientation), social resources (e.g., enabling peer relationships), and ecological ones (e.g., stable housing) mattered for the resilience of emerging adults who were exposed to adversity. Similarly, a more recent study of the resilience of a sample of SA emerging adults found that their resilience was rooted in psychological resources (e.g., hopefulness and tenacity), social resources (e.g., family support), and ecological ones (e.g., accessible recreation centres) [28].

In contrast, psychological and social resources are predominantly reported in the handful of published investigations of what enabled the resilience of emerging adults to COVID-19-related stressors. For example, a May 2020 survey of 825 emerging adults (median age: 20) in Belgium and Italy, showed that those who managed to maintain and even increase in-person contact with their social networks were disinclined to experience mental health care needs [17]. The researchers hypothesized that in-person contact was facilitated by household composition (i.e., residing with friends or family) and/or young people’s agency (e.g., willingness to contravene lockdown prohibition of in-person contact) [17]. Personal and social factors were also reported in the Zurich Project on the Social Development from Childhood to Adulthood [11]. For this study’s emerging adult sample, previous distress, COVID-19-related economic and lifestyle changes, and hopelessness were more strongly associated with distress than exposure to the virus itself. Social support, including maintaining contact with family and friends, enabled young adults to manage those stressors. Other useful resilience-supporting strategies cited by participants (e.g., maintaining daily routines; being physically active) are not universally available to young people, especially in contexts of strict lockdown [8].

Continuing in a similar vein, a survey with 898 young people (majority White or Asian; aged 18–30) in the United States during the early pandemic (i.e., April to May 2020) showed the protective value of relationships. Social support from family was particularly protective to young adults’ mental health. Whilst it was not specified whether protective family support was in person, virtual, or both, the researchers did note that support from peers or romantic partners was not similarly protective [16]. Likewise, a Chinese study with 2045 emerging adults (aged 18–25; 31.10% male) in late March 2020 (i.e., when the pandemic was already well controlled in China), reported that high levels of social support had enabling effects [29]. Again, family support was prominent, in that it was the only relational resource that apparently did not decline in the face of COVID-19.

### African emerging adult resilience to the COVID-19 pandemic

Despite the rising numerical dominance of young people in sub-Saharan Africa [30], they are vastly under-represented in the resilience to COVID-19 literature. An exception is a Nigeria-based study by Bada and colleagues [31]. They found that self-efficacy and emotional intelligence (both psychological resources) appeared to have significant resilience-enabling effects in a sample of 50 emerging adults located across Nigeria. Another exception was the study by Gittings and colleagues [8] with 12 emerging adults (aged 18 to 25) from two SA communities during South Africa’s strictest lockdown period (i.e., March 26 to April 30, 2020). The study underscored the negative material and psychosocial impacts of lockdown, including financial insecurity and an associated lack of necessities; disruptions to basic services; the frustration and fear of an uncertain future; fear of contagion; and psychological distress. Gittings and colleagues [8] make passing reference to participant resilience (i.e., information about how to manage contagion threats supported participants’ resilience).

In summary, the lack of attention to African emerging adult resilience to COVID-19-related stressors highlights the urgent need to report their seldom-heard insights. Thus, the objective of this article is to consider the resilience-enablers that supported 24 African emerging adults to manage the risks of later lockdown. From the perspective of a systemic resilience approach [20–24], resilience-enablers are likely to be found in emerging adults themselves (e.g., good health; personal agency) and in the systems that these young people are connected to (e.g., families; peer groups; community networks; the natural or built environment). They are also likely to be nuanced by situational and cultural factors.

## Methods

### Research paradigm

Given our interest in how 24 African emerging adults managed the psychological and economic risks of later lockdown (July 2020 –October 2020), we followed a phenomenological design and social constructivist principles. Phenomenological studies explore participants’ insights of a stated phenomenon and extrapolate commonalities and differences in those insights [32]. Our attention to participants’ understandings fits social constructivist reservations about universal truths [33]. It also fits the complexity (i.e., contextually responsive, multisystemic nature) of human resilience [23, 24], and the calls for resilience accounts informed by young people themselves [34].

### Researcher characteristics and reflexivity

We (the authors) have a track-record of using creative qualitative methods to better understand African and other participants’ understandings of resilience. Although we are all familiar with the context of South Africa (SA), DL—who was born and raised in SA—and MU reside in the Minority World (Global North). Further, we are all mature, privileged, white adults. Given the potential of these characteristics and experiences to influence the research process and shape our interpretations of participants’ insights, we contracted and guided two African emerging adults (Master’s students in Educational Psychology) to generate (BN) and co-analyse (BN; PK) the data. Both young people have first-hand experience of township life, also during the COVID-19 pandemic, and a sound understanding of systemic resilience (it was a module in their master’s studies). Neither contributed to the conceptual development of the study or this article (and therefore declined co-authorship).

### Context

In South Africa, COVID-19-related lockdown commenced on 26 March 2020. It was structured as five lockdown levels, with Level 5 enforcing the strictest measures [8]. From 1 June to 16 August 2020, Level 3 restrictions were enforced. This allowed non-essential businesses to open, but mandatory disease mitigation strategies (e.g., physical distancing; mask wearing in public); a curfew; delimited mobility; and limitations on social, educational, religious, or other gatherings continued. From 21 September to 29 December 2020, Level 1 prevailed. Disease mitigation strategies remained mandatory, but all other restrictions were less stringent (e.g., a later curfew; larger gatherings permitted). Thereafter, levels were adjusted upwards to manage South Africa’s 2^nd^ wave of infections.

Participants were residents of a township called eMbalenhle (eMba) in the municipality of Govan Mbeki. This township is the principal research site of the Resilient Youth in Stressed Environments (RYSE) study, South Africa [35]. SA townships, which are typically resource-constrained neighbourhoods, were designed to orchestrate racial segregation during the apartheid era [36]. In the democratic South Africa, they are still mostly inhabited by people of colour [37]. SA townships were disproportionately impacted by the social and economic challenges associated with the COVID-19 pandemic [38]. Even though the SA government instituted interim social relief measures (e.g., modest monthly grants; food packages), township residents generally did not manage to access these [38]. Furthermore, compliance with COVID-19 regulations is challenged by the densely populated, poorly serviced nature of township communities [39].

The adults living in Govan Mbeki mostly have low rates of education (only 31% have completed secondary school). Socio-economic disadvantage is widespread (e.g., 26% of residents are unemployed; 24% of households own a computer; 36% own a car; 62.3% report no internet access) [40, 41]. As in other parts of Govan Mbeki, and indeed SA [42], structural disadvantage and related protests (usually in response to poor service delivery and government corruption), characterise the RYSE-affiliated site [43].

Many families in the RYSE-affiliated site embrace traditional African values of respectful interrelatedness or Ubuntu [28, 43]. Ubuntu values emphasize that an African is not a rugged individual, but a person living within a community [44]. Generosity, tolerance, and spirituality are also associated with Ubuntu [45]. Whilst there are concerns that communal ways-of-being are being eroded [45, 46], those that remain true to its values anticipate material, psychological, and other support from their family and community, and reciprocate such support.

### Participants

As explained elsewhere [35], the RYSE Community Advisory Panel (CAP) successfully identified around 600 participants to participate in 2018–2020 research activities (including the study reported in the current article). For the purposes of the study being reported in this article, and in keeping with the modest sample sizes associated with qualitative research designs [47], the CAP was asked to recruit approximately 25 emerging adults from eMba who would be willing to share personal experiences of COVID-19-related stresses and how these were managed. The CAP identified 27 potential participants living in the RYSE-affiliated township. Because participant eligibility was governed by age (i.e., emerging adults only), a 17-year-old whom the CAP identified was excluded. The remaining 26 met the inclusion criteria (i.e., age, residence in eMba, willingness to share personal experiences). However, two were unable to follow through on their initial commitment to participate (i.e., *n* = 24). During the October follow-up, only one of the 24 July volunteers (i.e., Ayanda) was not available to participate.

#### Ethical issues pertaining to human participants

The research ethics committees of the Faculties of Health Sciences and Education at the University of Pretoria provided ethical clearance [UP17/05/01] as did the Social Sciences Research Ethics Committee at the University of Leicester (26759). All participants provided written informed consent and indicated whether their first name or a pseudonym should be used when making findings public. Participants were modestly compensated for their time and data-use.

#### Description of participants

As documented in Table 1, more young women (*n* = 14) than young men (*n* = 10) participated. The average participant age was 20. Participants’ households (mostly isiZulu-speaking) ranged in size from 1 to 14. Most participants (*n* = *16*) spontaneously disclosed knowing someone who had tested positive for COVID-19 and/or died from related complications. Nine were enrolled in college (*n* = 9); 7 were neither employed nor in education/training (NEET) but were actively searching for employment; 6 were high school students; and 2 were employees of local businesses. Only 2 participants (both young women) cared for children.

**Table 1:** Details of the sample.

Participant name	Sex	Age	Life circumstances @ start of lockdown	Acquainted with/related to someone who tested positive for/died from COVID-19
Ayanda	F	23	College student & part-time employed; household size: 8	Yes
Busisiwe	F	20	Not in employment, education or training (NEET); household size: 7	Not disclosed
Happiness1	F	24	College student; household size: 5	Yes
Happiness2	F	20	College student; household size: 4	Yes
Keletso	F	20	College student; household size: 6	Yes
Khumotso	F	19	NEET; household size: 5	Not disclosed
Lungelo	M	20	NEET; household size: 2	Not disclosed
Mamello	F	22	College student; household size: 7	Yes
Mikateko	F	24	College student; household size: 14	Not disclosed
Minky	F	21	NEET; household size: 5	Not disclosed
Naledi1	F	18	High school student & part-time employed; household size: 9	Yes
Naledi2	F	23	College student; household size: 6	Yes
Nkosinathi	M	21	High school student; household size: 4	Not disclosed
Sibusiso	M	24	College student & part-time employed; household size: 2	Yes
Sinethemba	M	24	Employed; household size: 5	Not disclosed
Siphiwe	M	22	NEET; household size: 6	Not disclosed
Sipho	F	23	NEET; household size: 6	Yes
Siyabonga	M	19	High school student; household size: 7	Yes
Tebogo	M	24	NEET; household size: 5	Yes
Thabang	M	20	High school student; household size: 1	Yes
Thabo	M	19	High school student; household size: 7	Yes
Tinyiko	F	21	College student; household size: 3	Yes
Tshegofatso	F	21	Employed; household size: 2	Yes
Willington	M	18	High school student; household size: 3	Yes

Not in Education, Employment or Training is abbreviated to NEET.

### Data generation and documentation

Given the dynamic nature of risk and resilience [20], data generation was iterative. It took place weekly in July 2020 (4^th^ month of SA lockdown) and again in the last week of October 2020 (7^th^ month of SA lockdown). The same participants were involved at both time points. Essentially, we added the October iteration to better understand if/how participant insights changed over time. Two methods (digital diaries and individual interviews) were used for both time points.

#### Digital diary methods

Diary studies enable new insights into participants’ habits and perspectives in day-to-day environments and social contexts [48], sometimes termed ‘in situity’ [49]. *Digital* diaries specifically have been found to offer a novel, context specific way of understanding the everyday lives of young people [50–52], facilitating rich data collection through multiple forms of media, immediacy, and participant freedom to amend/edit or pre-record their contributions [53]. Even in contexts of chronic structural disadvantage, young people’s lives are typically mediated by their presence in a digital world. As such, viewing their experiences through a digital lens is important to building meaningful understandings of their experiences of/responses to COVID-19-related stressors.

Following Alaszewski [54], we invited a digitally-mediated, regular, personal and contemporaneous record of an individual’s perspectives and experiences. Put differently, every 2–3 days we asked participants to share their perspectives and experiences of COVID-19-related challenges and how they managed them. Based on work completed by Janssens et al. [51], we anticipated that this frequency would minimize recall challenges without burdening participants. The following prompts guided participants’ digitised reflections: (i) What COVID-19-related challenges or stresses did you experience in the past 2 or 3 days?; (ii) How did you manage these challenges or stresses?; and (iii) Who or what helped you to manage these challenges or stresses? Except for the specific reference to COVID-19, these prompts were similar to the resilience-focused questions used in preceding resilience studies with young people exposed to significant stress [35].

**Fig 1:**
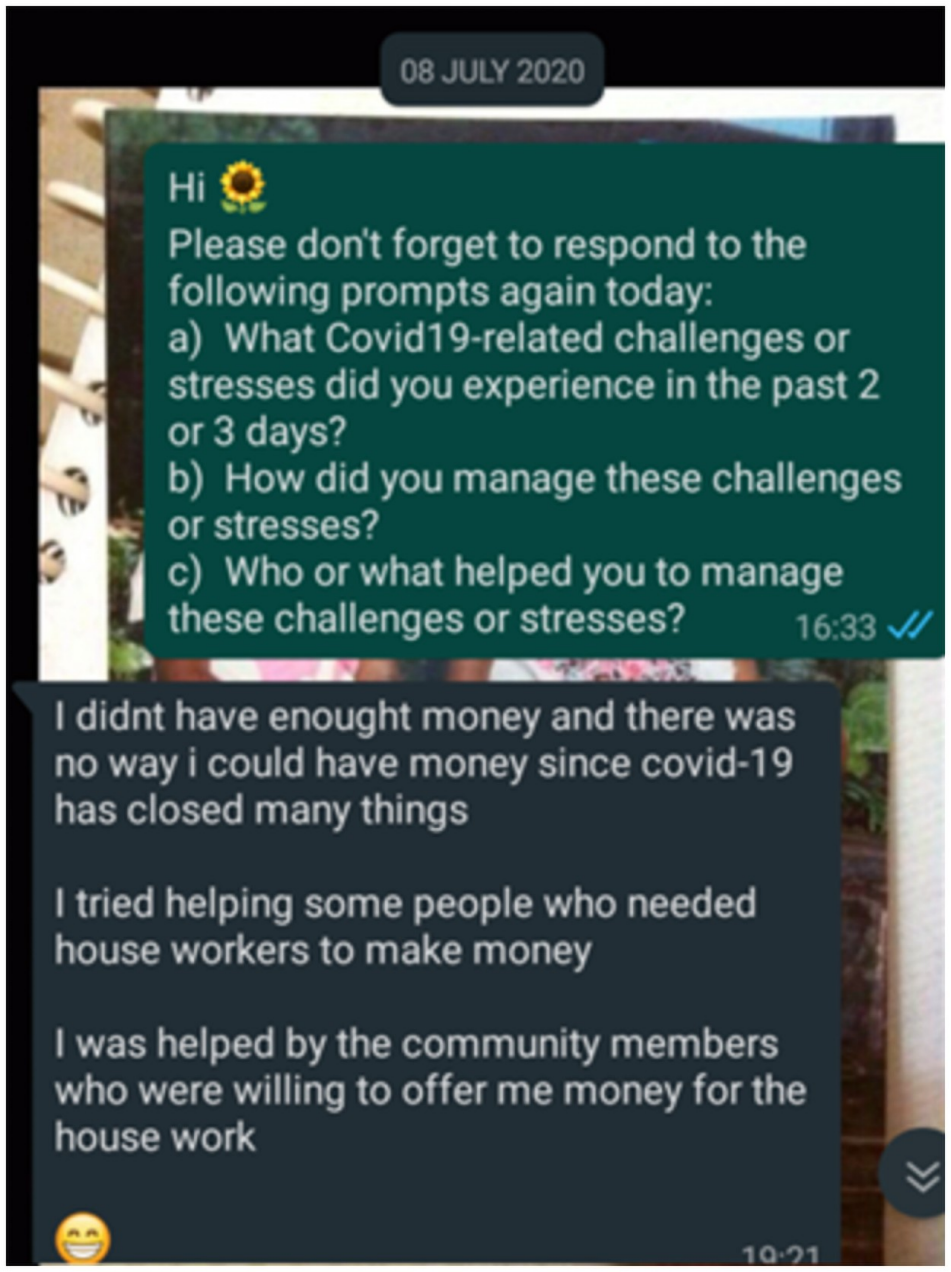
Diary entries were typically brief.

Responses could be textual, auditory, or visual and could be shared (as per participant preference) via Short Message Service (SMS), Moya^™^ message, or WhatsApp^™^. Neither visual nor auditory responses were popular (three participants included two visual images each; two participants sent a total of four voice notes). Instead, participants generally favoured text messages (worded in English). Regardless of modality, all participants chose to share all diary entries via WhatsApp^™^. Very occasionally, some participants used SMS interchangeably with WhatsApp^™^. During the July data generation period, 21 participants provided at least two diary entries per week; the remaining three did so for three of the four weeks only (2 sent none in the first week; 1 sent none in the third week). Although BN reminded participants to share diary entries, she did not coerce participants if they sent none. Excepting for Ayanda, all participants provided at least two diary entries in the last week of October when we followed up. Most diary entries were brief (see Fig 1 for an example).

Participants sent their diary entries to a study-dedicated telephone number (and associated WhatsApp^™^ account) that was managed by BN on a password-protected device. BN acknowledged receipt of all diary entries. She transferred the textual and visual diary entries via secure cloud storage to LT and DL; DL stored the data in the University of Leister’s secure file drive. BN transcribed the voice notes; they were similarly transferred and stored.

#### Semi-structured telephonic interviews

BN engaged participants in semi-structured interviews. To do so, she telephoned them weekly during the July data generation period at a time of their choosing and audio-recorded the conversation. She also telephoned them at a time of their choosing during the last week of October to do a follow-up interview. Although telephonic interviews have their limitations (e.g., rapport is challenged), they fit with COVID-19 restrictions on movement and social interaction. Further, the richness, quality, and depth of data are retained through this modality [55].

During the telephonic interview, BN asked participants what COVID-19-related challenges they had experienced during the preceding week, how they managed those challenges, and who (if anyone)/what (if anything) supported that management. On average, interviews lasted 30 minutes. Because these interview questions and digital diary prompts were very similar, the digital diary and interview content overlapped. Still, the interview data were more detailed as BN could probe when responses were vague (e.g., can you tell me a little more about that?) Because she was fluent in isiZulu and English, and familiar with Sesotho/isiXhosa, participants were free to converse in any of these languages. For the most part they spoke a mixture of English and isiZulu. Interviews lasted between 20 and 60 minutes. BN transcribed the recordings verbatim and, where necessary, translated into English. BN transferred the transcripts via secure cloud storage to LT and DL; DL stored the data in the University of Leister’s secure file drive.

The translations were independently verified by a mother-tongue isiZulu speaker. She listened to the original recordings, compared them with the English transcripts, and made isolated, negligible changes. DL, who is isiZulu literate, moderated the transcripts.

### Data analyses

ATLAS.ti^™^ software was used to manage the data. BN and LT conducted an initial analysis of the data (i.e., diary texts and interview transcripts). To do so, they followed Creswell and Creswell’s explanation of an inductive, thematic content analysis approach [47]. That entailed reading and rereading the data to identify segments that answered two broad questions: what COVID-19-related challenges were reported, and how did participants mange those challenges? Working separately, BN and LT extracted relevant segments and assigned a provisional label that paraphrased their content. LT then compared these provisional labels and found that they were very similar (i.e., wording varied but meaning did not). She combined them to form a list of codes denoting challenges (e.g., limited employment opportunities; insufficient funds for basic household expenses) and ways of managing reported challenges (e.g., asking for help; extended family fund travel expenses; neighbours share food). She then used these codes to generate initial themes [56]. To that end she grouped similar codes and relabelled them thematically to reflect patterns in the data (e.g., limited employment opportunities and insufficient funds for basic household expenses both constituted livelihood threats; neighbours share food and church provides for material needs represented community co-facilitation of emerging adult capacity to manage COVID-19 stressors). Each initial theme was defined in terms of inclusion and exclusion criteria. Thereafter, PK independently extracted data that fit the thematic coding frame, as per their inclusion and exclusion criteria. The fact that her extraction matched the data that had informed the thematic code groups (i.e., initial themes) encouraged our confidence in the thematic analysis.

Following Braun and Clarke [56, 57], LT then reviewed and further developed the themes. For instance, she combined initial themes that were risk-specific and resilience-specific to illustrate how specific risks elicited specific resilience-enablers. She was attentive to how contextual dynamics (e.g., township realities; traditional African values) played into the risks and resilience-enablers that young people reported. While such sensitivity could bias analyses, it typically supports a more nuanced understanding and is therefore encouraged in thematic analyses [57]. This refinement resulted in a thematic interpretation of the data that fit better with current multisystemic understandings of resilience as a dynamic process that draws on multiple resilience-enablers and is responsive to risk and context [21–23].

### Trustworthiness

The iterative process of data generation supported a dataset that was rich and thematically saturated, meaning that further data collection would be unlikely to challenge key findings or generate new insight [57, 58]. Its richness was advanced by BN’s capacity to converse with participants in their mother tongues and elicit detailed responses. Although multiple coders are not a requirement for trustworthy thematic analyses [57], our confidence in the analyses of this rich dataset was buoyed by coder consensus (i.e., the similarly in the BN’s and LT’s provisional codes) and PK’s extraction of data matching the data that had informed the initial themes. Saldana [59] explains that consensus, which can be determined conversationally (as was done in our study), is associated with trustworthy analyses. In addition, the multi-author team offered opportunity for external auditing of the analyses: the authors who were not directly involved in the data analysis, reviewed the raw and coded data and confirmed the proposed findings. Addressing Morse’s concerns about member checking [60], participants were not invited to review the findings. Instead, as done with preceding qualitative RYSE work [35], CAP members were invited to consider the findings (i.e., LT conversed with them about the key findings that were identified). They confirmed them.

## Findings

**Fig 2:**
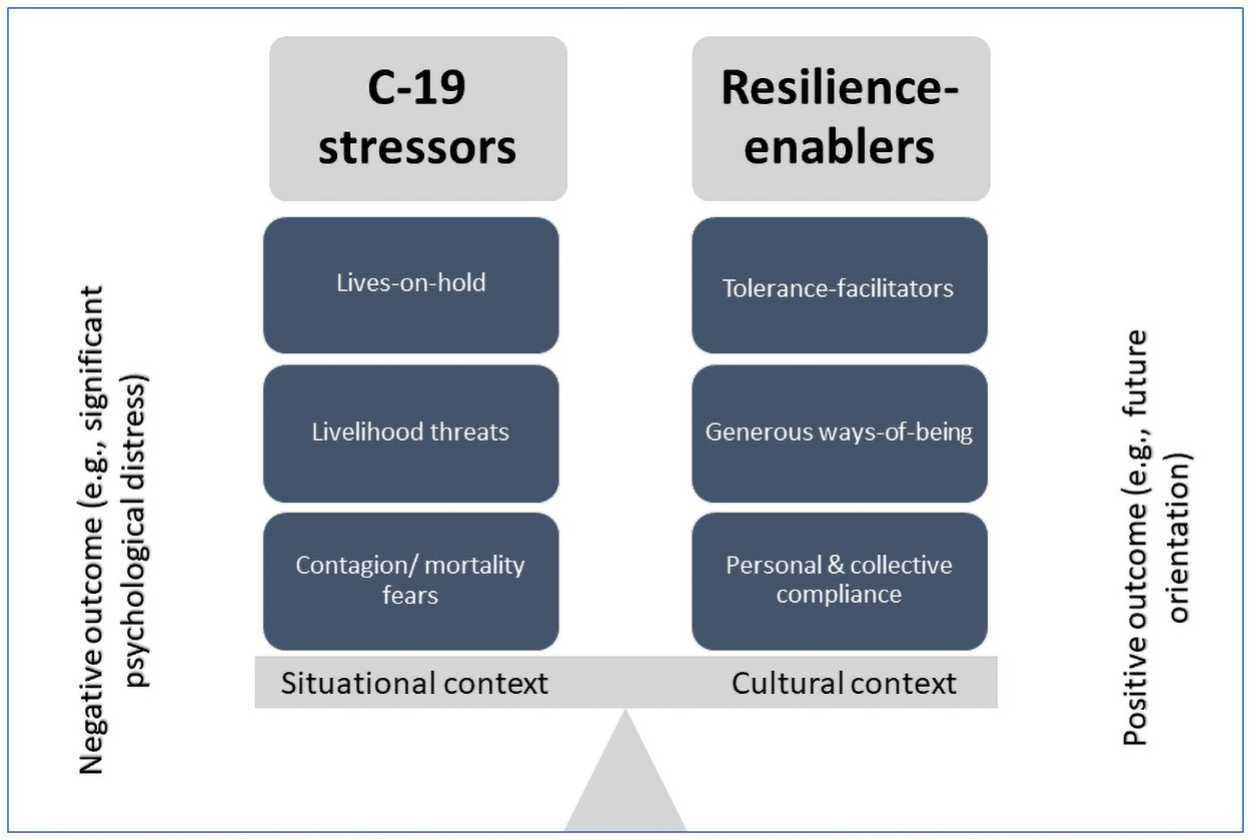
Summary of findings. Management of COVID-19-related stressors (that were strongly informed by the structurally disadvantaged context of eMba township) was supported by resilience-enablers (that were strongly informed by traditional African values that promote interdependence and tolerance). To tip the balance board in favour of positive outcomes would require redress of the structural drivers of the COVID-19 stressors that were prominent in participant accounts.

Participating emerging adults living in eMba experienced the pandemic as stressful. In July and October, the prominent stressors took the form of contagion and/or mortality fears, livelihood threats, and lives-on-hold. In July and October, resilience to these stressors was variably enabled, with the more prominent resources being personal and collective compliance, generous ways-of-being, and tolerance-facilitators. eMba’s situational and cultural context—i.e., structural disadvantage; interdependent ways of being-and-doing—informed young people’s experience of COVID-19 stressors and their management thereof. In Fig 2 these findings are summarised using the metaphor of a balance board.

### Personal and collective compliance with disease mitigation strategies eases contagion/mortality fears

Participants feared contracting COVID-19. They feared experiencing the respiratory complications associated with COVID-19. They worried that COVID-19 infection would lead to death. For instance: “[shop] had to close due to two cases of Corona in their staff … this brought stress to me … what if I was infected?” (Mamello, Week [W] 1 diary). “I’m so afraid of COVID-19. People are suffering [struggling] to breathe, people are feeling pains in their bodies… Dying like this is not on my wish list” (Lungelo, W3 Interview); “What will happen to me if I’m infected… since COVID also kills, will I survive, or will I be part of those that die?” (Naledi1, W4 Interview); “I was literally worried that I might have it [COVID-19] or [that] I’m dying” (Sibusiso, October follow-up interview).

To manage these fears, participants took control over their (potential) exposure to the virus by committing to the government-mandated disease mitigation strategies. For instance, Nkosinathi said, “I can see that it won’t be easy for this thing to infect me because I follow the rules, wherever I am. I have a mask and sanitizer. I keep my distance” (W2 Interview). Sinethemba was similarly confident: “the core prevention measures—if I can do that, then I think I am safe from harm” (W4 Interview). Sipho’s October follow-up digital diary entry about how he was managing COVID-19 challenges included “listening to my family that I should … follow those safety precautions or regulations”.

**Fig 3:**
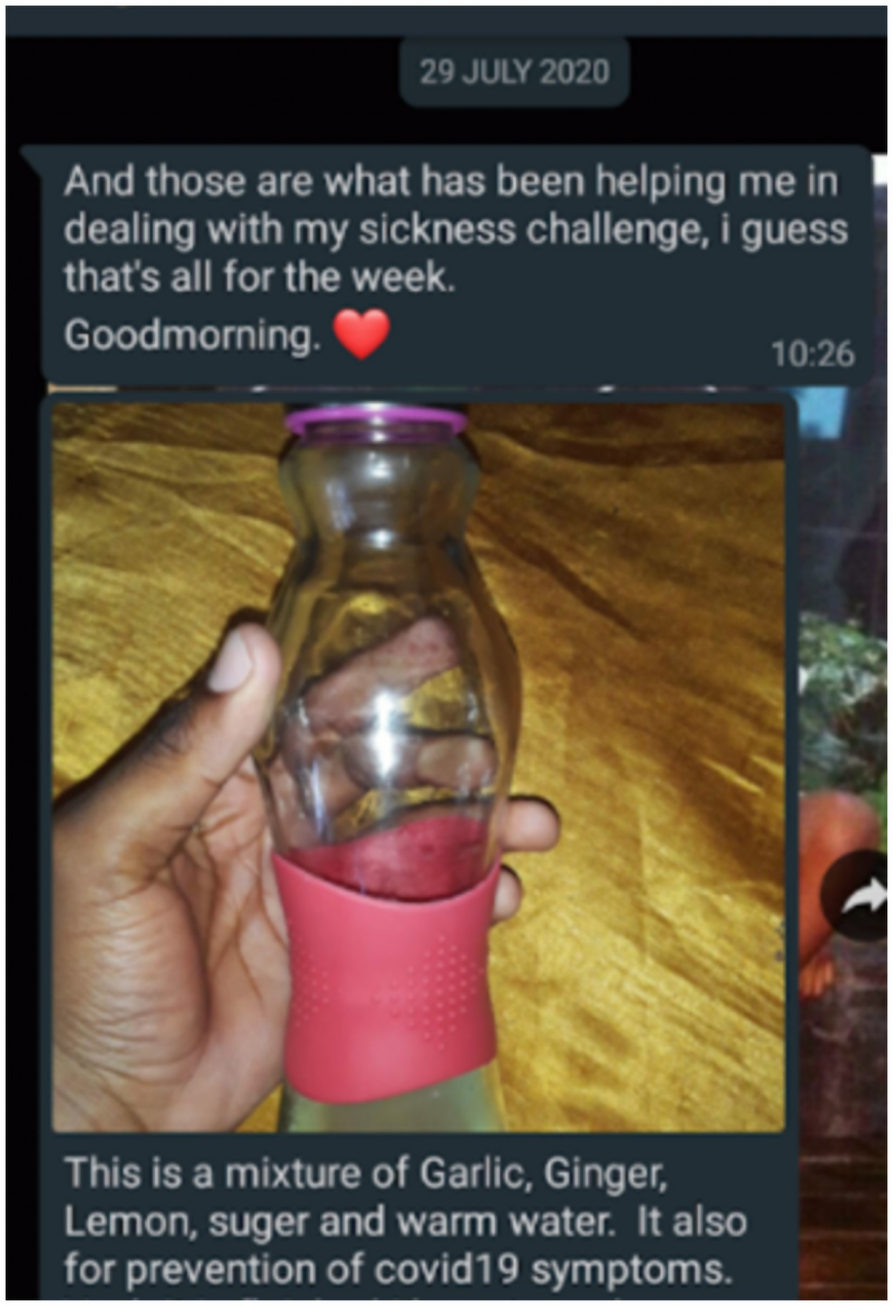
Lungelo’s W4 digital diary entry illustrating traditional medicine.

Taking control also lay in using traditional remedies and trusting their curative power (see Fig 3). Mostly, this entailed drinking homemade brews comprising herbs, spices, and/or plants traditionally believed to have medicinal value:

We are helping ourselves by drinking … garlic and ginger …[and] umhlonyane [Artemisia Afra] and it helps a lot … when I drink those things, I have this hope that this thing will not infect me, that even if it does infect me, it will be hard for it to kill me

(Sipho, W2 Interview)

My mom tells me to get umhlonyane [Artemisia Afra] and flu remedies … that makes me feel safe. Like, even if I notice the slightest symptoms, there are remedies

(Tshegofatso, W3 Interview)

However, personal commitment to COVID-19 regulations or traditional remedies was seldom enough to allay fears. When households and communities were also compliant, participants experienced a greater sense of control over contagion/mortality threats. Lungelo said, “When I see people that are wearing their face masks…then it’s a relief that, OK, maybe here I’m safe. Even though I’m not 100% safe, but then at least I am safer” (W2 Interview). Participants also believed that witnessing a collective of compliant people would have a positive knock-on effect for all: “If I wear a mask and sanitize and keep my distance, at least I’m protecting the next person. So, the next person can see that the one in front is protecting me; I should protect the one behind… it’s a chain.” (Happiness 1, W4 Interview)

Structural constraints—particularly disrupted service delivery and reliance on public transport—complicated personal and collective compliance. Locals who were sceptical about the reality of the pandemic (i.e., “Thomases” who needed to “see it to believe it”; Keletso, W1 Interview) and thus disinclined to follow COVID-19 safety measures, added to these complications:

Some don’t believe because they say this thing is for whites, people who are rich, and people who travelling different countries …most of the time they don’t wear masks and this puts us at risk because we don’t know if they are infected or not and they can infect us also. It is difficult because I feel I am not safe. I am afraid of getting sick anytime.

(Thabo, W1 Interview)

We usually experience water cuts—like today, there was no water—this thing of us washing our hands, we can’t do it properly. There was a strike [about the water and electricity supply disruptions]. There were many people. My brother was also there. There was no social distancing. They were not wearing masks. So, that thing stressed me because we don’t know who has COVID-19 … He could have brought it home, and then we would all be affected

(Busisiwe, W2 Interview)

**Fig 4:**
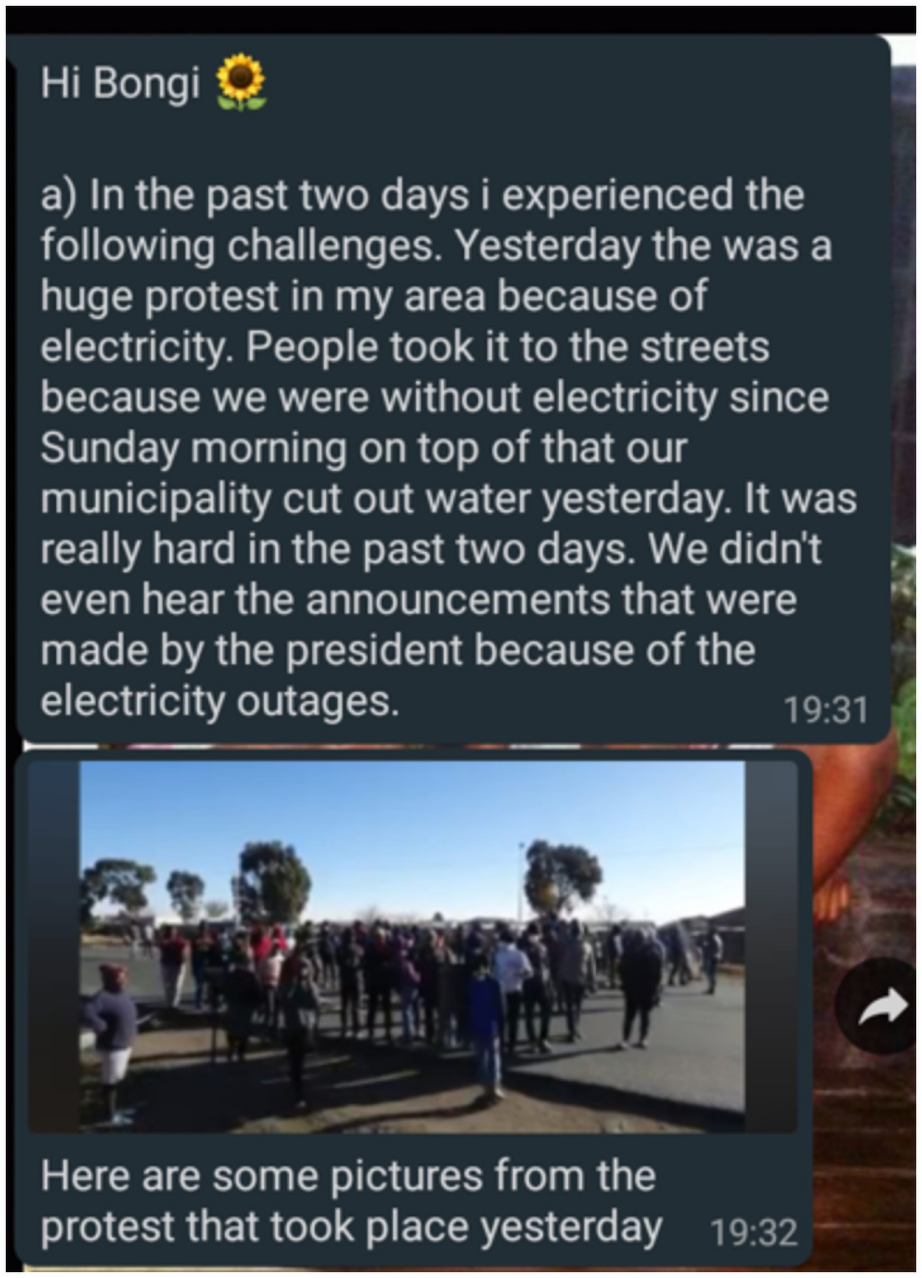
Minky’s W2 digital diary entry.

The electricity has been on and off … plus, it’s winter—it’s very cold—so, it was difficult for people to stay home and to keep warm at home … [so] most of the people were doing something that was wrong. It’s like a social gathering: they were making fire using a “mbawula” where they use a drum, and then they add tyres and coal and then they keep warm using that. …[usually] it’s more than 20 people sitting there [around the “mbawula”] and going back home very late

(Minky, W2 Interview; also see Fig 4)

It is really bad in the taxis … I took a taxi and there were these other women who just didn’t want to listen. They didn’t want to wear their masks …

(Sipho, W4 Interview)

When household members and neighbours returned from social gatherings or crowded venues/events, some participants urged COVID-19 regulation compliance. Siphiwe reported: “I sat down with my neighbour and explained to them how serious Corona is; I can see that they are now wearing their masks” (W3 Interview). Supporting others to be compliant not only helped participants feel personally safer, but also emotionally rewarded: “You have this courage that at least there’s a difference I’ve made in a life. You can also be okay in your spirit that at least there’s something I have done”. (Tebogo, W3 Interview).

Whilst there was some reference to formal regulation of personal and collective compliance (e.g.,. “I had forgotten my mask and I wasn’t allowed inside the mall because the [security] guard said I’m a threat without a mask.” Sibusiso, W4 Interview), exposure to local COVID-19 health impacts diminished COVID-related scepticism and galvanised compliance. A more compliant collective allayed participants’ contagion fears:

Now, it’s quite rare to find someone who does not believe that corona is real because it is evident that it kills… all of a sudden so-and-so is no longer alive due to COVID

(Tinyiko, W4 Interview)

They’re no longer together in the streets; they’re no longer playing soccer like before. They are sitting indoors. That actually makes me happy. At least we won’t make each other sick… It helps me to be calm, because if more people are staying safe, then there are less chances of me being infected

(Keletso, W4 Interview)

Some participants expressed concern that less stringent lockdown levels (like Lockdown Level 1 that was in place in October 2020) were associated with a decrease in collective compliance. For instance, Tinyiko was frustrated because “now even the police are relaxed. They are also not wearing masks so they cannot stop you and remind you of the mask” (October follow-up interview). Similarly, Happiness1 said:

In Level 5 at least they [community] were complying. So, now with Level 1, it’s like we are all relaxed. Everyone is relaxed. It’s like they don’t care. They have that belief that COVID is no more, although it’s still there; it’s just that now it’s like low or minimized. So, they are now ignorant unlike when we were in Level 5 … everyone is doing as they please, they don’t care whether you are without your mask, whether you get into the taxi and you’ve sanitized, they no longer care

(October follow-up interview)

### Generous ways-of-being buffer livelihood threats

All the participants experienced and/or witnessed the pandemic’s capacity for material deprivation:

It is going to be a disaster …already people are hungry now. Even at the malls, these boys have started stealing people’s plastic bags [shopping] because they are hungry

(Ayanda, W4 Interview)

Corona is killing most of our business [roadside food stall] and it’s hard for us… most of the people lost their jobs… they were the ones who were supporting us…that means there is no food [for us] at home, no money for transport. Sometimes I have to hike to go to school

(Mikateko, October follow-up interview)

To survive, participants searched/continued to search for part- or full-time employment. Sometimes their agency was rewarded: “I got myself a piece job: I’ll be helping people with their laundry so that I am also able to make a living” (Naledi1, W1 Interview). Mostly, though, participants reported failed job searches: “I’ve been struggling with job hunting” (Lungelo, W2 Interview); “Employment is scarce” (Thabo, W4 Interview); “I have not found a job and I have been looking” (Siphiwe, October follow-up interview). Likewise, there was scant reference to dependable government or other formal supports:

The government just needs to keep their promises that they made. Remember, they promised people R350 [approximately $23] and some people didn’t get that. They also promised food parcels, but some people have not received them and those that got them, only got them for 2 months … A lot of places do not have water—the government was supposed to install water for those people. That is something that is essential during this time because they have to always wash their hands

(Busisiwe, October follow-up interview)

**Fig 5:**
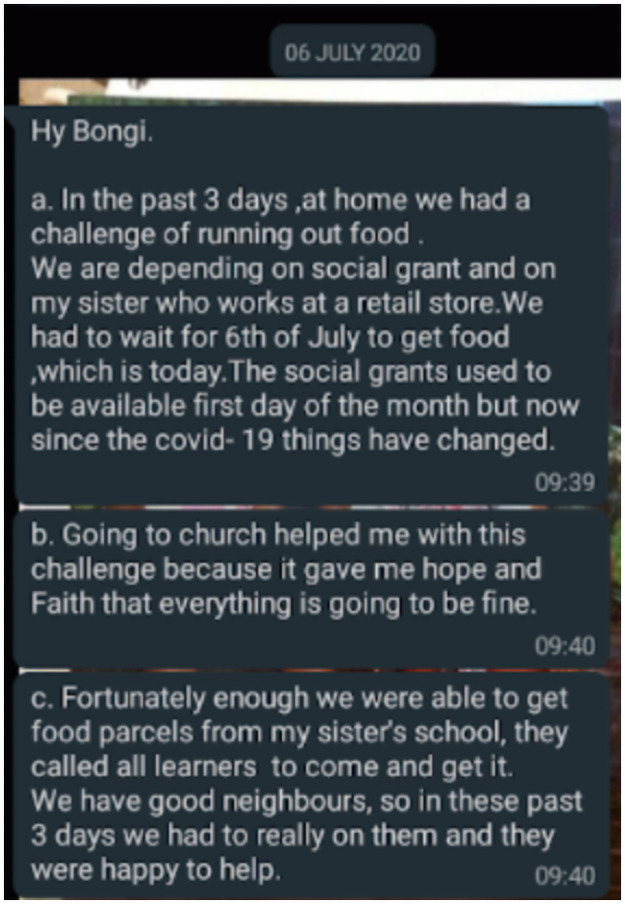
Busisiwe’s W1 digital diary entry.

In contrast to the infrequent and/or sceptical references to formal supports, informal social networks were integral to the management of livelihood threats (see Fig 5). Family, friends/acquaintances, neighbours, teachers, and faith-based organisations could be relied on to share material resources and provide emotional support. Their selflessness translated into a dependable safety net:

I know we are going through a difficult time as a country, but we still have *Ubuntu*. We still think about the next person

(Happiness2, W2 Interview)

She [Aunt] told me, ‘You can’t just drop out of school because of this thing [uncle’s illness and related emotional and financial stress]. We are here, we will deal with it. We will make a plan … you need to not worry’ … Her words just lifted me

(Keletso, W3 Interview)

When I stand by the stop sign where the taxi passes, I usually run into people that I know and then I ask for a lift and they give me a lift

(Thabo, W2 Interview)

Besides sitting down with friends and discussing to see if it would be possible to maybe get together and help those families who have been affected, we have other sisters [community members] that we have spoken to, who have also promised that they will support like with food parcels … so at least there’s something we are doing to show that ‘you guys are not alone, we are all in this together’

(Tebogo, W4 Interview)

My church really did play a big role during lockdown because they were giving out food parcels and stuff. So, we know that with these basic things—mealie-meal, rice, fish, oil, eggs—we could plan around it

(Minky, October follow-up interview)

At the end of the day, you realise that everybody is living in fear, but they are making it through … So, it is definitely the people around me who helped me through these challenges

(Tshegofatso, October follow-up interview).

### Tolerance-facilitators make lives-on-hold bearable

All participants lamented that pandemic-related challenges had disrupted their routines and dreams:

I might not graduate this year, apparently. Even though I may complete, I’m not excited because there won’t be any graduations, because all graduations are stuck

(Happiness 1, W2 interview)

I was expecting things to happen. Now those things aren’t able to happen due to the fact that we are faced with this Corona … I feel like I’m stuck with my life

(Khumutso, W4 Interview)

We need to … get things back to normal so that we can continue with life. Right now, life is at a standstill

(Willington, W4 Interview)

I guess there is nothing that will go back to the usual normal

(Happiness2, October follow-up interview)

Keeping busy facilitated participant tolerance of pandemic-related disruptions and related helplessness, frustration, and other negative emotions. To keep busy, they engaged in faith-based activities, were active on social media sites, played/listened to music, or exercised. Because COVID-19 regulations restricted the use of the local recreation centre, safe open spaces or roads with sidewalks were valuable spaces for exercise:

I keep myself busy. I read the Bible, or I go online, go to Facebook, check the jokes, anything that can distract me

(Ayanda, W2 Interview)

Keeping busy somehow gives closure to me that this is the best you can do. You are not Superman, you don’t have any superpowers. If I had them, I would have long ago helped the whole world to go back to normal. So, it gives me closure that at least there’s something you’re doing, you’re not just sitting and feeling helpless all the time

(Happiness2, W2 Interview)

**Fig 6:**
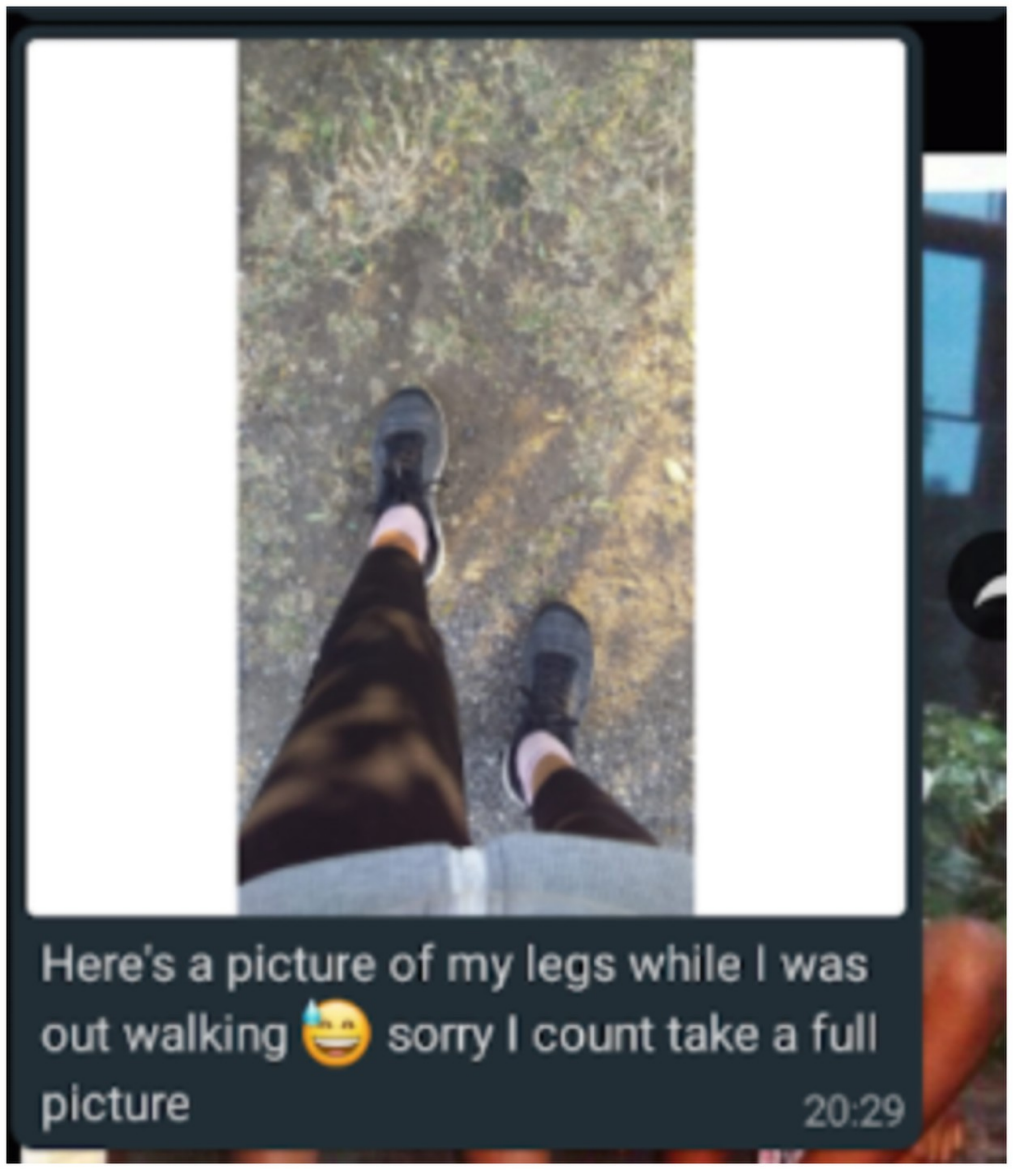
Minky’s W3 digital diary entry.

I started jogging. So, it’s sort of replacing gym … At least if I go for a walk or if I go jogging, I come back feeling a lot better and my mind is a little more relaxed

(Minky, W3 Interview; also see Fig 6)

Some participants reported that it was easier to keep busy during lock-down periods with less stringent rules, such as Lockdown Level 1 that prevailed during the October iteration of our study. To illustrate:

School was closed, going to the shops and going to see friends—everything was at a standstill. The only thing that we were busy with was our phones; the only thing that you could have access to. But, as things go on and the levels decrease, it makes things easier for me. It is easier because I’m able to go to school … I am able to just decide let me go for a breath of fresh air at the mall…

(Mamello, October follow-up interview)

Now it is [Lockdown] Level 1, I can socialise. I can go to events, I can go to the gym, the mall. So, I can see people, and people can come and see me

(Thabang, October follow-up interview)

Tolerant meaning making also supported participants to manage the frustration of disrupted routines/dreams. Hope inspired by their faith-based community/beliefs and knowing that others were also experiencing COVID-related stasis made the disruptions easier to tolerate, as did the example or advice of tolerant others:

I strongly believe that when God is on our side, everything is fine even though this pandemic is there

(Tebogo, W1 interview)

COVID-19 is here to stay. You have to embrace it, and then you move on. Stressing about it won’t help

(Tinyiko, W2 Interview)

People are accepting COVID-19, that it’s something we are going to live with … so, them doing it constantly, without them losing hope, shows that people are accepting it and then I just have to accept it as well and just move on

(Mamello, W3 Interview)

I also like praying a lot. That gave me a little comfort … I told myself that I will give everything to God, He will sort it out

(Keletso, October follow-up interview)

I just had to accept the situation and understand that right now we are faced with COVID-19 and we are not the only ones that are suffering

(Busisiwe, October follow-up interview)

## Discussion

The purpose of this article was to report how 24 African emerging adults managed the psychological and economic risks of lockdown in a SA township during July and October 2020 (i.e., month 4 and 7 of South Africa’s protracted lockdown). Their personal accounts illuminated a lockdown-reality characterised by a fear of contagion, threatened livelihoods, and a sense that life was on-hold. Such challenges complicate emerging adult developmental tasks, including completing education or training, establishing a career, committing to a long-term partner, and achieving functional independence [6]. Pre-existing studies of the impacts of the COVID-19 pandemic on emerging adults, albeit mostly during early lockdown in non-African countries, noted these same challenges [7, 11, 16, 17, 19], as did a single South African, early lockdown study [8]. Apparently, these challenges do not abate in later lockdown as our participants identified them in the fourth and seventh month of SA’s lockdown.

As explained by emerging adults themselves, resilience to lockdown-related challenges drew on personal and collective compliance, generous ways-of-being, and tolerance-facilitators. These resilience-enablers implicated resources from multiple systems, including psychological, social, and built environment ones. Importantly, these resilience-enablers and associated resources were reported in both July and October. This longevity suggests that emerging adult resilience to COVID-19 stressors requires multisystemic supports that are dependable and durable. Interestingly, in the context of eMba, less stringent lockdown levels had ambiguous implications for the resilience-enablers young people reported. Greater freedom of movement and fewer restrictions meant that it was easier for some young people to keep busy, but also raised concern about declining community compliance with COVID-19 regulations.

Whilst the identified resilience-enablers implicated resources from multiple systems, the informal social system was particularly salient. That salience overlaps with non-African studies that emphasized the importance of friends and family to emerging adult resilience to COVID-19 stressors [11, 16, 17]. At the same time, the prominence of informal social connections to emerging adult resilience should not eclipse the role of emerging adult agency in their continued connectedness during lockdown conditions. Their capacity to reach out to and value others fits their period of development [6]. The fact that they were still doing so in the fourth and seventh month of lockdown extends the appreciation for the importance of personal agency to emerging adult resilience reported in early lockdown studies [8, 17].

As theorised [20, 21, 23], contextual factors could account for which systemic resources mattered to a greater/lesser extent. In contexts of resource constraint, such as townships, it is not unusual for South African young people to under-report built/natural environment resources or formal supports [61], also in COVID-challenged times [3, 8]. Given this, it was not surprising that participant reference to protective built/natural environment resources was mostly implicit (e.g., how young people kept busy implicated outdoor spaces to exercise). As also reported by Gittings et al. [8], formal supports (e.g., effective public health campaigns) could be glimpsed in young people’s knowledge of how to protect themselves against COVID-contagion. In contrast, participant accounts explicated that a disabling built environment (e.g., cold houses) jeopardized locals’ compliance with COVID-19 regulations. Similarly, young people spelled out that their capacity to manage COVID-related stressors was confounded by an unreliable service ecology and a government that apparently reneged on its social relief commitments. Again, this pattern persisted over the two time points of the study. This context of perennially inaccessible and/or inadequate environmental and formal supports makes one wonder whether generous ways-of-being and tolerance-facilitators were default resilience-enablers. Whilst generosity and tolerance are values associated with Ubuntu [44–46], it is plausible that accessible, relevant, and reliable formal supports would have lessened emerging adults’ reliance on culturally valued ways-of-being. Still, respect for and enactment of collectivist social values appear to have protective potential for individuals challenged by the COVID-19 pandemic. Subsequent cross-cultural studies would be helpful to gauge their protective value in societies that typically favour individualism, as would studies seeking to understand how collectivist values might be advanced in the interests of limiting COVID-19-related stress and promoting vaccination uptake [62].

Contextual dynamics are also implicated in the salience of a compliant collective to participants’ accounts of resilience. The township situation—i.e., a densely populated, service-poor space with limited opportunity for physical distancing [8, 38, 39]–likely amplified fears of contagion and livelihood threats and accentuated the importance to personal and communal health and peace-of-mind of everyone being compliant. In this crowded context, resilience required sustained enactment of collectivist social values. Interestingly, in this context there was little evidence of resilience being advanced by limiting exposure to news about infection rates and COVID-19-releated deaths [26]. Instead, exposure to the lethal potential of COVID-19 infections galvanised compliance with government-mandated and other disease mitigation strategies, thereby advancing self and other protection.

### Actionable implications

The resilience-enablers (i.e., personal and collective compliance; generous ways-of-being; tolerance-facilitators) were mostly consistent over the four-month period of our study. There was some indication that changes in lockdown level and related restrictions had ambivalent value for some of the resilience-enablers young people identified. As restrictions eased and people sensed life was returning to ‘normal’, collective compliance was threatened but tolerance-facilitators—specifically keeping busy—were enabled. It will be important for legislators to proactively manage the complex potential of the stringency of a lockdown level to facilitate certain resilience-enablers and constrain others.

For the most part, the identified resilience-enablers were scaffolded by personal strengths and caring connections to family, friends, and community members. They were also informed, albeit to a lesser extent, by built and natural environment resources and public health campaigns. As such, emerging adult resilience to COVID-19-related challenges may not be explained mono-systemically (e.g., as only/mostly psychological strengths [31]). Similarly support for emerging adult resilience to COVID-19 stressors should transcend an emphasis on relational resources within the family system [11, 16, 17], and/or transient supports. Our study therefore suggests the fundamental importance of multisystemic approaches to evaluating, preventing, and coping with the anxiety, stress and fears surrounding COVID-19 [21–23], including specific culturally nuanced norms and values. Interventions that solely target families, for example, are unlikely to take necessary account of the protective and risk factors at play as young people travel from adolescence into adulthood. While interim supports (e.g., emergency relief funds) can and do enable resilience, interventions ideally need to facilitate consistent access to every day supports (like caring connections and safe spaces to exercise).

Moreover, built and natural environment resources need to feature more strongly. Their absence in accounts of emerging adult resilience to COVID-19-related stressors [11, 16, 17, 31] and/or implicit role (as in our study), may not detract from their potential value to human resilience, particularly in times of lockdown. In short, advancing emerging adult resilience to COVID-19-related challenges will require a multisystemic approach.

Further, emerging adults’ capacity to adjust to COVID-19-related challenges requires multisystem resources that are not random. Instead, these resources must be responsive to a given situational and cultural context [20, 21, 23], and themselves capable of adaptation. In densely populated contexts, like that of our study, support for resilience to COVID-19-related stressors must include culturally compatible ways of reducing scepticism/conspiracy theories and galvanising mutual responsibility for limiting contagion. Whilst over-attention to the health of significant others could undermine individual wellbeing [18], our study invites researchers and practitioners to consider to how local COVID-related infection/fatalities could be used to advance local compliance with COVID-19 disease mitigation strategies. At the very least, participants’ experience that awareness of rising local infections/deaths advanced collective compliance, cautions against ready uptake of advice to limit exposure to COVID-19 news to support personal resilience [26]. Likewise, in under-resourced contexts like that of our study, emerging adult resilience to COVID-19 stressors hinges on improved services and social relief measures [8], as well as opportunities for decent employment. Civil society is key to fast-tracking emerging adult access to these much-needed resources [3]. In addition, equitable access to built and natural environment resources (e.g., safe indoor/outdoor spaces) should be expedited, particularly when young people’s immediate environment stymies opportunities to comply with disease mitigation strategies or tolerate the frustrations of disrupted routines or dreams. Essentially, in resource-constrained contexts, continued emerging adult resilience to COVID-19 stressors requires a social justice imperative.

Finally, emerging adult capacity to contribute to their own resilience and that of others must be recognised and championed. In the current study, emerging adults were effective public health agents (e.g., spontaneously cautioning their immediate social networks about the value of disease mitigation strategies). Extending formal opportunities for emerging adults, particularly unemployed ones, to co-facilitate their social ecologies’ management of COVID-19 contagion risks will offer young people an additional way to keep busy in COVID-disrupted times. To further manage livelihood threats and facilitate emerging adult developmental goals, their public health agency should be rewarded (e.g., salaried).

### Limitations

To facilitate rapid data gathering, eligible participants were recruited by the RYSE CAP. A more public recruitment (e.g., via social media) might have prompted inclusion of young people with different views and mitigated the potential for bias. Likewise, there are limitations as to claims we can make about whole populations based on our findings. Nevertheless, our focus was on ensuring trustworthiness of our key messages.

We inferred participant resilience from their four-month commitment to our research study and from their employment-focused agency and/or engagement in education (see Table 1). In the face of the relentless challenges that characterised their lives, we believed that such commitment and agency constituted evidence of normative functioning [20, 28]. More objective proof of normative functioning might be preferable in future studies.

While we added the October iteration to better understand if/how the resilience-enablers that supported management of lockdown risks changed over time, we acknowledge the brevity of this period. Had the period been extended, it is very possible that we would have discerned changes in what participants reported as resilience-enabling [63]. Our findings were similarly limited because we did not formally investigate how young people’s life circumstances had changed (or not) for the better/worse over the course of the study period. Given that COVID-19-related stressors have persisted beyond the October 2020 iteration of our study and that novel resilience-enablers have been introduced since then (e.g., vaccine access), it would be valuable to investigate COVID-19-related changes in emerging adults’ lives and how these inform risk and resilience.

## Conclusion

Our article illuminates what a sample of South African emerging adults believed to support resilience in the face of COVID-19 stressors experienced during lockdown in July–October 2020 and draws attention to how situational and cultural context inform both risk and resilience. It highlights the importance of redressing structural drivers of COVID-19-related distress and of sustaining resilience-enabling collectivist social values. Reporting these young people’s insights redressed the historic inattention to African emerging adult resilience [28], and contributed a contextually congruent, later lockdown account to what was known about emerging adult resilience to (mostly early lockdown) COVID-19-related stressors [7–9, 11]. Although many countries have succeeded in vaccinating at least 10% of their population and reducing pandemic-related stress, many African countries have not yet been able to do so [64]. Until vaccine rollout is equitably fast-tracked and COVID-related threats to emerging adult wellbeing are better managed, attention to the resilience of this young population remains non-negotiable.
